# Ascites

**Published:** 1854-03

**Authors:** 


### *Ascites—Endermic Treatment.*

Dr. Falcot recommends fomentations, with decoction of digitalis. Two ounces of digitalis are boiled in a quart of water down to a pint: compresses, dipped in the decoction, are laid over the abdomen, and covered with oil silk. The kidneys are soon powerfully affected.—*Phil. Med. & Surg. Jour.*

If true, this is a fortunate prescription.

---

*Vaccination.*—Now is the time to prevent the Small Pox. This loathsome and fatal disease is on the increase in our Eastern Cities. Let the *Western* prevent its communication and spread by vaccination.

